# Fabrication of Polyamide-6 Membranes—The Effect of Gelation Time towards Their Morphological, Physical and Transport Properties

**DOI:** 10.3390/membranes12030315

**Published:** 2022-03-10

**Authors:** Mohammad Ebrahimi, Wojciech Kujawski, Kateryna Fatyeyeva

**Affiliations:** 1 Faculty of Chemistry, Nicolaus Copernicus University, 7 Gagarina Street, 87-100 Toruń, Poland; mohammad.ebrahimi@doktorant.umk.pl; 2 Polymères Biopolymères Surfaces (PBS), Normandie University, UNIROUEN, INSA ROUEN, CNRS, 76000 Rouen, France; kateryna.fatyeyeva@univ-rouen.fr

**Keywords:** polyamide-6, gelation time, porous membrane, phase inversion method, hydrodynamic water flux, pore compression, membrane hydrodynamic permeability

## Abstract

Porous polyamide-6 membranes were fabricated via a non-solvent induced phase inversion method, and the influence of gelation time on the properties of the membranes was investigated. Membrane samples with various gelation times were prepared. The evaluation of the membranes’ properties was carried out by various analyses and tests, such as scanning electron microscopy, atomic force microscopy, contact angle, wet and dry thickness, mean pore size measurements, porosity, water uptake, mechanical resistance, hydrodynamic water fluxes, membrane hydrodynamic permeability, and retention testing. The scanning electron microscopy images (both surface and cross-section) demonstrated that the increase in gelation time from 0 (M_0_) to 10 (M_10_) min led to the morphological change of membranes from isotropic (M_0_) to anisotropic (M_10_). The wet and dry thickness of the membranes showed a downward tendency with increasing gelation time. The M_0_ membrane exhibited the lowest bubble contact angle of 60 ± 4° and the lowest average surface roughness of 124 ± 22 nm. The highest values of mean pore size and porosity were observed for the M_0_ sample (0.710 ± 0.06 µm and 72 ± 2%, respectively), whereas the M_10_ membrane demonstrated the highest tensile strength of 4.1 MPa. The membrane water uptake was diminished from 62 to 39% by increasing the gelation time from 0 to 10 min. The M_0_ membrane also showed the highest hydrodynamic water flux among the prepared membranes, equal to 28.6 L m^−2^ h^−1^ (at Δp = 2 bar).

## 1. Introduction

Porous membranes are mainly classified into three categories: microfiltration, ultrafiltration, and nanofiltration membranes [1,2]. Therefore, the mean pore sizes of porous membranes lie between 0.2 nm and 10 µm [1,3]. Based on the separation process, different operating pressures are needed for porous membranes [4,5,6]. The driving force for the aforementioned processes is created by a pressure difference, meaning that by applying sufficient pressure onto the feed stream, separation will be occur [5,7]. Today, porous membranes are utilized in various fields such as the food, car, biotechnological, and electronic industries in order to separate a wide range of bacteria, yeast cells, macromolecules, colloids, viruses, aerosols, and smoke particles [2,3,8,9,10]. In general, the filtration process for porous membranes can be carried out in two modes: dead-end and cross-flow [11,12]. Although dead-end filtration is quite common, the fouling phenomenon occurs during the separation owing to the deposition of particles on the surface and/or in the membrane bulk [13,14]. Consequently, the membrane shows a resistance for passing the flow and eventually the permeate flux is substantially reduced [1,5]. In this research, dead-end filtration was used to investigate the hydrodynamic permeability of polyamide-6 (PA6) membranes in contact with pure water. With the aim of obtaining porous membranes with reasonable physical and chemical properties, several improvements have been invented including the use of nanoparticles (NPs) [15], application of inorganic compounds [16], graft polymerization [17], modification of the surface via polymer immobilization [18], plasma surface modification [19], and unconventional radiation methods [20]. In fact, the main aim of these methods is an increase in the flux and selectivity, and a decrease in the fouling of porous membranes during the separation process [15,16,17,18,19,20]. Furthermore, a wide range of factors can improve membrane performance, such as the nature of the polymer, solvent and non-solvent, the composition of the casting solution, the composition of the coagulation bath, the gelation and crystallization behavior of the polymer, the location of the liquid-liquid demixing gap, the thickness of the casting knife, the rate of membrane casting, the temperatures of the casting solution and coagulation bath, and the gelation time [1,5]. These factors can considerably change the morphology of membranes and, thus, their performance [21,22]. For instance, Li et al. [23] investigated the influence of membrane thickness on the morphology of membranes. The polyethersulfone and polyimide membranes were made by the phase inversion method. It was found that membrane thickness had a direct influence on the morphology of the membranes, as the thickness enhancement changed the membrane structure from sponge-like to finger-like. In another study, Fan et al. [24] studied the influence of the coagulation media on the morphological features of a polyamide membrane. The polyamide membranes were elaborated by the phase inversion method using various types of coagulation media, such as water, methanol, ethanol, and *n*-propanol. It was found that various types of coagulation media provide membranes with various morphology, mechanical stability, and porosity [24]. See-Toh et al. [25] investigated the effect of membrane formation parameters including evaporation time (10 s, 30 s, 50 s, 70 s, and 24 h), the concentration of polymer (polyimide), and thermal annealing (at 100, 150, and 200 °C) on membrane performance. It was found that by increasing the polymer concentration and evaporation time, the toluene flux through membranes at 30 bar decreased considerably. The membrane flux was also reduced with rising annealing temperature. Additionally, the polymer concentration, evaporation time, and annealing temperature changed the morphology of nanofiltration membranes. Paulsen et al. [26] studied the impact of evaporation time on the morphology of cellulose acetate membranes. The membranes were obtained by a phase inversion method in which the cast polymer solution was kept in the atmosphere for a specified time and then submerged in the coagulation bath. It was found that the evaporation step had a strong influence on the creation of macrovoids. Vandezande et al. [27] prepared polyamide membranes via the phase inversion method. The influence of the polymer concentration, solvent and non-solvent ratio, evaporation time, and the composition of coagulation bath on membrane properties was studied. In the case of an increase in the polymer concentration from 15 to 25 wt% the rejection of the membrane reached 100%; however, the permeance reduced. Furthermore, by enhancing the solvent content from 0 to 20 wt%, the permeance decreased, whereas the rejection of the membranes increased. The same trend regarding the permeance and rejection of the membrane was observed by raising the evaporation time for polyamide membranes. The increase in evaporation time provokes a decrease in the size and number of macrovoids. Moreover, various non-solvent baths were used, such as water, methanol, ethanol, propanol, and isopropyl alcohol, and the membrane obtained in a methanol non-solvent bath showed the highest flux [27].

In this work, PA6 membranes were prepared by a non-solvent induced phase inversion method, and the main objective of this research was to investigate the influence of gelation time on membrane morphology and properties. Scanning electron microscopy (SEM), atomic force microscopy (AFM), contact angle (CA), membrane thickness (T_w_ and T_d_), water uptake (WU), mean pore size (MPS), porosity (ε), mechanical stability, hydrodynamic flux of water, membrane hydrodynamic permeability (L_p_), and retention testing were investigated during the characterization of porous PA6 membranes.

## 2. Experimental Section

### 2.1. Materials

The pellets of PA6 possessing a specific weight equal to 1.4 g cm^−3^ and a melting point of 230 °C were purchased from the ZWCH STILON S.A. (GorzówWielkopolski, Poland). Formic and acetic acids were provided by Chempur (Piekary Śląskie, Poland). Calcium chloride was purchased from Sigma-Aldrich (Poznań, Poland). Reverse osmosis-purified water was used in the experiment.

### 2.2. Membrane Fabrication

**Figure 1:**
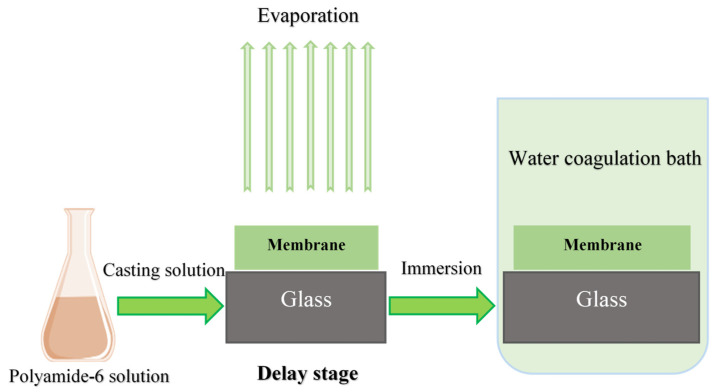
Schematic of preparation of PA6 membrane by phase inversion technique.

**Figure 2:**
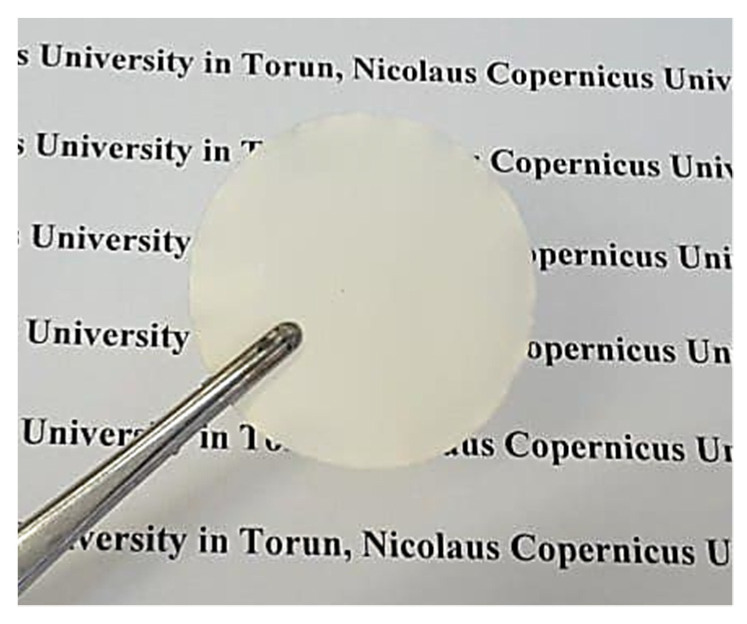
Image of obtained PA6 membrane.

The PA6-based solution was prepared according to the method proposed by Ceynowa and Adamczak [28]. A mixture of following compositions: 52.4 wt% formic acid, 8.3 wt% acetic acid, 8.3 wt% calcium chloride, and 17.5 wt% distilled water was prepared, and subsequently 13.5 wt% of PA6 pellets was added to this solution. Then, the polymeric solution was stirred for a specified time. The PA6 membranes were elaborated by solution casting in which the polymer solution was cast onto a glass plate (neat and dry) by an automatic film applicator (Erichsen Gmbh Co., Hemer, Germany), with the determined casting rate (10 mm s^−1^) and the slit of casting knife equal to 0.2 mm (Figure 1). The cast PA6 solution was exposed to air at the ambient temperature for a specified time, called the gelation time (Table 1). Then, a glass plate was immersed into a coagulation bath [27,29]. The membranes were placed in reverse osmosis water for 24 h in order to remove the solvent residues. The obtained membranes were of white color, flexible, and opaque (Figure 2).

## 3. Characterization of PA6 Membranes

### 3.1. Scanning Electron Microscopy

The morphology of the porous PA6 membranes (surface and cross-section) was determined by SEM analysis (SEM, Quanta 3D FEG, FEI, Prague, Czech Republic). Furthermore, the thickness of the membranes was also measured by SEM. Prior to cross-sectional analysis, the membrane samples were cryofractured in liquid nitrogen.

### 3.2. Atomic Force Microscopy

Atomic force microscopy was used to evaluate the surface roughness and topological morphology of the PA6 membranes. The topography of the membrane’s surfaces was investigated via microscopy with a scanning SPM probe of the NanoScope MultiMode type (Veeco Metrology, Inc., Santa Barbara, CA, USA). To determine the surface roughness of the membranes, a tapping mode was used. The scanned area for each sample was 5 µm × 5 µm. The data acquisition and processing was conducted using Nanoscope v6.11 software (Bruker Optoc GmbH, Ettlingen, Germany). The roughness average (Sa) and root mean square (Sq) were measured by AFM. The roughness average is the most common factor which is used for surface roughness measurement. In general, detection of general variation is done via this parameter and it is calculated by the following equation [30].

(1) $$\mathrm{Ra}=\frac{1}{L_{x}L_{y}}\int_{0}^{Lx}\int_{0}^{Ly}|f(x,y)|dxdy$$

where

f(x,y): surface relative to the center plane,
L_x_ and L_y_: dimensions of the surface.

The standard deviation of the profile height is presented as the root mean square and is calculated by the following equation [30].

(2) $$Rq=\sqrt{\frac{\sum {\left(Z_{i}-Z_{ave}\right)}^{2}}{N_{p}}}$$

where

Z_i_: the current height value,
Z_ave_: the average height,
N_p_: number of points in a given area.

### 3.3. Thickness Measurements

The thickness of wet and dry membrane samples was determined by micrometer (Sylvac, Switzerland). The thickness was measured 30 times at different places over each membrane sample to evaluate the accuracy of the membrane formation. The thickness of the wet membrane (T_w_) was measured after the process of phase inversion in a coagulation bath. Then, the membranes were put into an oven at 50 °C for 24 h and their dry thickness was measured again (T_d_).

### 3.4. Contact Angle Measurement

The membrane hydrophilicity was evaluated by contact angle (CA) measurement. For this purpose, a goniometer (Biolin Scientific, Gothenburge, Sweden) was used. Generally, there are two main methods for CA measurement: sessile drop and captive bubble methods. Sessile drop technique (CA measurement by a liquid) is the most common technique for quantifying the surface wettability of a solid substance [31]. In this method, CA is described as a formed angle by a liquid where liquid/gas phases meet the solid phase. Generally, there are three main theories used for determination of the contact angle using Young’s, Wenzel’s, and Cassie–Baxter’s equations [32].

One of the fundamental approaches uses Young’s Equation (3) for the description of the wetting process; however, with this equation, only the wettability of smooth surfaces can be determined [32,33].

(3) $${\cos\theta }_{Y}^{}=\frac{\gamma^{\mathrm{sg}}-\gamma^{\mathrm{sl}}}{\gamma^{\mathrm{lg}}}$$

where θ_Y_, γ^sg^, γ^sl^, and γ^lg^ are the contact angle, interface tension of solid/gas, solid/liquid, and liquid/gas, respectively. Low values of θ_Y_ (less than 90°) indicate a hydrophilic surface in which the liquid spreads on the surface and makes it wet, while high values of θ_Y_ (greater than 90°) demonstrate a hydrophobic surface indicating that the liquid is unable to wet the solid surface.

The Wenzel Equation (4) is obtained by introducing the roughness factor (r) to Young’s equation. The roughness factor is defined as the actual area on the membrane surface divided by the apparent geometry area, and, since the actual area on the membrane surface is always larger than the apparent geometry area, the roughness factor is always greater than 1 [32,34].

(4) $${\cos\theta }_{W}=\frac{r\left(\gamma^{\mathrm{sg}}-\gamma^{\mathrm{sl}}\right)}{\gamma^{\mathrm{sl}}}{=\mathrm{rcos}\theta }_{Y}$$

Therefore, when the θ_Y_ is greater than 90°, the roughness factor will increase the hydrophobicity, whereas when the θ_Y_ is lower than 90°, the roughness factor will increase the hydrophilicity.

The Wenzel equation was modified for hydrophobic surfaces by Cassie and Baxter, as shown in Equation (5) [32].

(5) $${\cos\theta }_{\mathrm{CB}}{=r\varphi \cos\theta }_{Y}+\varphi +1$$

where the φ is the horizontal projection area fraction of the solid-liquid interface.

However, to measure the contact angle of super hydrophilic and porous membranes, the captive bubble method is more accurate than sessile drop, because in the sessile drop method, the water droplet will be absorbed by the super hydrophilic and porous surface immediately, and consequently, measuring the equilibrated CA is practically impossible [31,35]. To measure the CA via captive bubble method, the membrane is first immersed in the liquid (water), then the air bubble is formed on the membrane by a special type of needle (“J” type needle). In this research, reverse osmosis water was used as a liquid. The measured CA in this method is described by Equation (3) [31,35]. One Attention software (version 4.02) was used for data acquisition and processing. In this study, the bubble contact angle of PA6 membranes was measured at 24 ± 3 °C and 45% RH.

### 3.5. Water Uptake (WU)

The membrane water uptake reveals the membrane’s ability to absorb water molecules. Prior to the measurements, membrane samples were placed in an oven at 100 °C for 6 h. Then, samples were weighed (W_dry_) and submerged in deionized water at an ambient temperature for 24 h. The surface water was removed using tissue and the membrane was weighed again (W_wet_). The values of water uptake for PA6 membranes were calculated using Equation (6). The measurements were repeated three times for each sample to estimate the measurement accuracy [36,37].

(6) $$\mathrm{Water}\;\mathrm{uptake}\;(\%)=\mathrm{WU}(\%)=\left(\frac{W_{\mathrm{wet}}-W_{\mathrm{dry}}}{W_{\mathrm{dry}}}\right)\times 100$$

### 3.6. Mean Pore Size and Porosity Measurements

The mean pore size, and maximum and minimum pore size of PA6 membranes were measured by Porometer 3G Micro (Quantachrome Instruments, Boynton Beach, FL, USA) using the modified bubble point method [5,38,39]. This method is based on measuring the required pressure to pass an inert gas (e.g., nitrogen) through the wet membrane [40]. Prior to the measurement of the bubble point, the membrane was wetted with a liquid possessing a low surface tension (e.g., Porefil with the surface tension of 16.6 mN m^−1^) in order to fill all the pores of the membrane [5,39,41].

**Figure 3:**
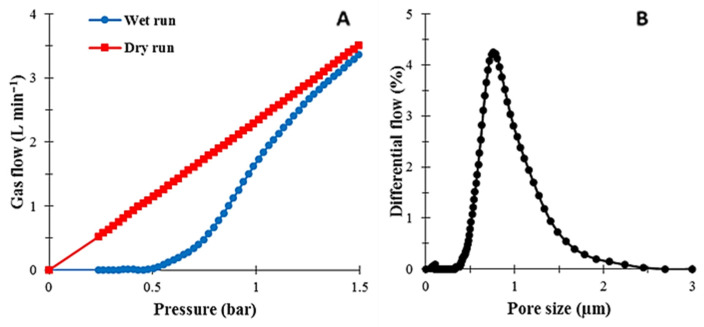
Results of the bubble point method for the PA6 membrane: (**A**) gas flux in wet and dry runs; (**B**) pore size distribution.

During the “wet run”, the inert gas starts to remove liquid from the wet membrane and in the “dry run”, the gas flow is measured through the dry membrane in which all the pores are open (Figure 3A). The Laplace equation (Equation (7)) shows the correlation between the pressure and the pore radius [5,39]. With the pressure rising, pore size measurement is started. Generally, the membrane transport properties are significantly affected by the pore size and pore size distribution (Figure 3B). The measurements were repeated twice for each sample to estimate the measurement accuracy.

The membrane pore radius can be calculated using the Laplace Equation (7).

(7) $$r_{p}=\frac{2\sigma }{\Delta p}=\cos\theta$$

where r_p_, σ, Δp, and θ are the pore radius, the surface tension of air-liquid interface, the applied pressure, and the wetting angle with membrane solid matrix. It can be assumed that cosθ = 1 if the liquid wets the membrane. Moreover, the porosity of the PA6 membrane was measured by the following equation [36,42]:

(8) $$Porosity(\%)=\varepsilon (\%)=\left(\frac{W_{wet}-W_{dry}}{\rho_{water}\times A\times L}\right)\times 100$$

where W_wet_, and W_dry_ are wet and dry membrane weight (kg), respectively, ρ_water_, A, and L are the water density (kg m^−3^), the membrane surface area (m^2^), and the membrane thickness (m). The membrane porosity was measured three times for each membrane to estimate the accuracy of measurement.

### 3.7. Mechanical Resistance

The mechanical resistance of a polymer membrane is an essential element for the separation processes. The influence of gelation time on mechanical properties was investigated by tensile analysis. The mechanical stability was measured by utilizing the Shimadzo EZ-X machine (SHIMADZU, Kyoto, Japan) and the data analysis was conducted via Trapeziumx software (version 1.5.4). This analysis was conducted according to the PN-C-89034:1981 standard. The membrane was cut in standard form (30 mm and 5 mm, length and width, respectively) and placed between two clamps. Then, the membrane sample was broken at a 1 cm min^−1^ strain rate. The analysis was carried out at 25 ± 2 °C, and 40 ± 2% of RH. The mechanical tensile strength was measured five times for each membrane sample [43,44].

### 3.8. Membrane Filtration Performance

**Figure 4:**
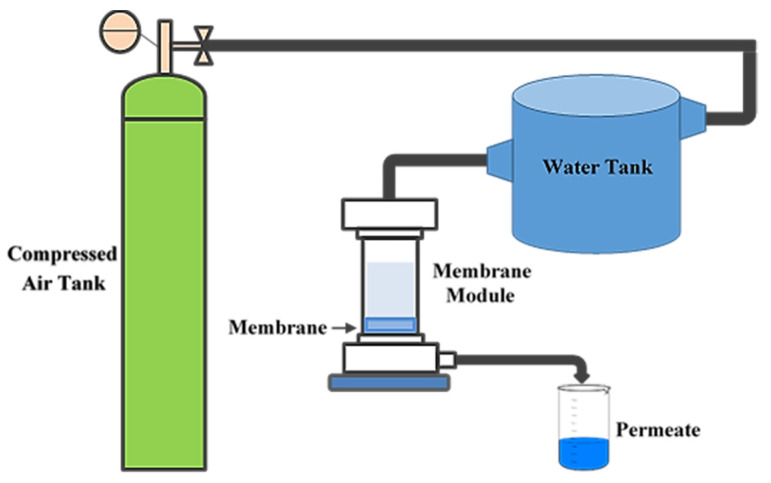
MF experimental setup.

A dead-end experimental setup was utilized (Figure 4) to measure the hydrodynamic water flux of the PA6 membranes. This setup contained a cylindrical cell made of Plexiglas, possessing a volume of 200 mL and an active surface area of 25.2 cm^2^. Prior to filtration, the PA6 membrane was cut to fit the cell size and placed in the bottom of the cell. A rubber gasket was used for sealing, then, the cell was filled with pure water. A water tank with a volume capacity of 5 L was used to allow continuous measurements over a longer period of time. Moreover, a compressed air cylinder was utilized to apply the required pressure (driving force) for water transport. In this research, three operating pressures (0.5, 1.0, and 2.0 bar) were used to evaluate the hydrodynamic water flux of the membranes with different gelation times, according to Equation (9):

(9) $$J_{v}=\frac{\Delta V}{A\cdot \Delta t}\;$$

where J_V_ is the hydrodynamic flux of water (L m^−2^ h^−1^), ΔV is the permeate volume of water (L), A is the membrane area (m^2^), and Δt is the operation time of the filtration (h).

The membrane hydrodynamic permeability (L_p_) is calculated from the Hagen–Poiseuille Equation (10) [5]:

(10) $$J_{v}=\frac{{\varepsilon \pi r}^{2}}{8\eta \tau }\cdot \frac{\Delta p}{l}\;$$

where

(11) $$L_{p}=\frac{{\rho \varepsilon \pi r}^{2}}{8\eta l\tau }$$

wherein ρ is the fluid density (kg m^−3^), ε is the membrane porosity (m^2^), r is the radius of pore (m), η is the viscosity of the medium (bar hr), l is the membrane thickness (m), τ is the tortuosity factor, L_p_ is the membrane hydrodynamic permeability (L m^−2^ h^−1^ bar^−1^), J_V_ is the hydrodynamic water flux (L m^−2^ h^−1^), and Δp is the operating pressure (bar).

Combining Equations (10) and (11), the following expression for L_p_ can be derived:

(12) $$L_{p}=\frac{J_{v}}{\Delta p}$$

Retention testing was also conducted to evaluate the impact of gelation time on polyethylene glycol (PEG) retention. PEG with the molecular weight of 20,000 Daltons was selected for this test. In order to minimize the effect of concentration polarization, the feed solution was stirred at a rate of 1000 rpm during the separation process. Prior to retention testing, each membrane was compressed at 3 bar for 2 h, then the test was conducted at a 2-bar operating pressure. The retention of the PA6 membranes was calculated by the following equation.

(13) $$R=1-(\frac{C_{p}}{C_{f}})$$

where C_p_ and C_f_ are the PEG concentrations in the permeate and feed solution, respectively. PEG concentrations were measured by using the UV—VIS Spectrometer Lambda 25 apparatus.

## 4. Results and Discussion

### 4.1. Scanning Electron Microscopy

**Figure 5:**
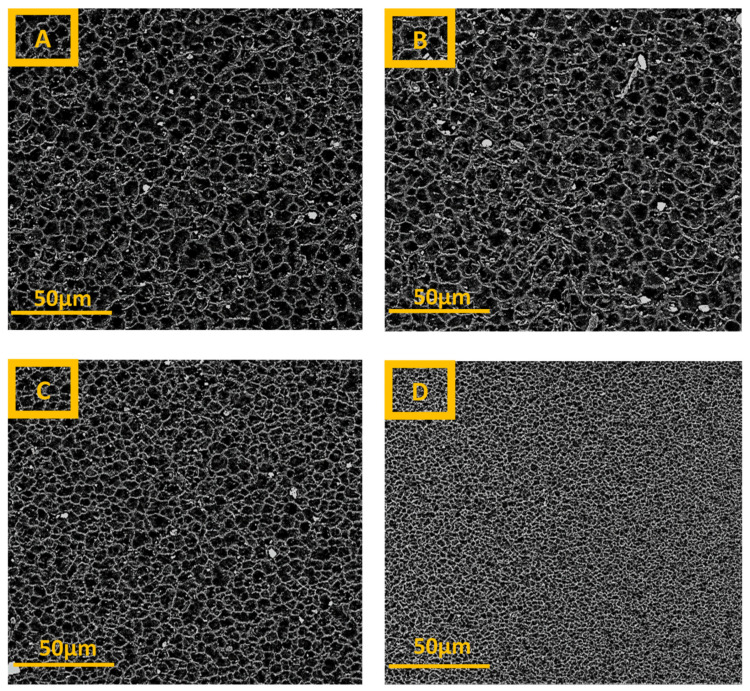
The SEM images with magnification of 1500× for PA6 membranes with various gelation times: (**A**)—0 min; (**B**)—2 min: (**C**)—4 min, and (**D**)—10 min.

**Figure 6:**
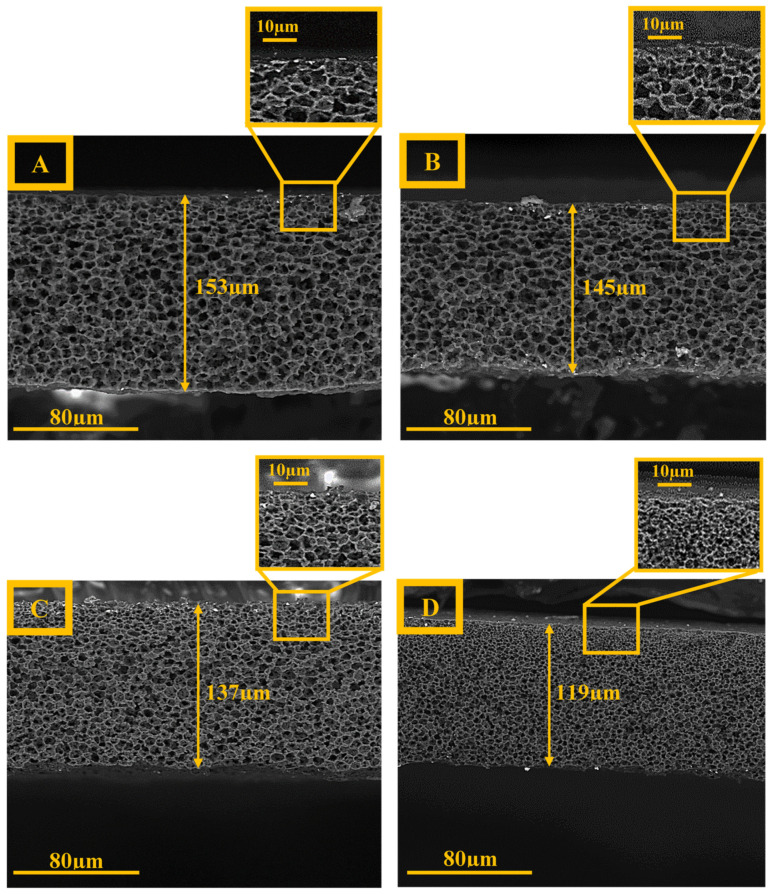
The cross-section SEM images (1000×) for PA6 membranes with different gelation times: (**A**)—0 min; (**B**)—2 min: (**C**)—4 min, and (**D**)—10 min.

SEM images of the surfaces of the porous PA6 membranes with various gelation times are shown in Figure 5. The images demonstrate that the increase in gelation time from 0 to 2 min does not change significantly the membrane’s apparent morphology, as the pore size of the M_2_ membrane is slightly smaller than that of the M_0_ membrane. A further increase in gelation time (to 4 and 10 min) leads to the formation of more compact (denser) membranes with smaller pores and, consequently, the M_10_ sample possesses the smallest pores. Furthermore, from the cross-sectional SEM images (Figure 6), it can be seen that up to 2 min of the gelation time, the membranes have an isotropic morphology. However, with further increases in gelation time, the morphology of the membranes changed from an isotropic to an anisotropic one. Moreover, the cross-sectional images show that a skin layer with smaller pores is obtained at the top part of each membrane and this layer becomes denser with smaller pores with the increasing gelation time. Such behavior can be explained by the fact that during the enhancement of the gelation time from 0 to 10 min, a larger amount of the most volatile solvent (formic acid) evaporated from the casting solution. Therefore, changes in the skin layer of the M_10_ membrane are more visible than those in the M_0_ membrane.

### 4.2. Atomic Force Microscopy

The information regarding the surface roughness of the resultant membranes with various gelation times are shown in Table 2 and Figure S1. Both values regarding the roughness average (R_a_) and the root mean square (R_q_) demonstrated (Table 2) that with the enhancement of gelation time, the surface roughness of the PA6 membranes increased. Generally, the distance between the peaks and valleys in the surface of the membrane determines the surface roughness of that surface, and the larger the distance between these (peaks and valleys), the rougher the surface obtained. It can be observed that the surface roughness of the M_0_ and M_2_ membranes is the same. In fact, the exposure time of the cast polymeric solution with air for the M_2_ sample is not considerable, therefore, the morphology of the M_2_ membrane is same as the M_0_ (Figure 5, Figure 6 and Figure S1). With further increases in the gelation time, membranes with more roughness were obtained and the highest surface roughness was reported for the M_10_ sample (R_a_ = 189 ± 53 and R_q_ = 240 ± 59). Since the M_10_ membrane was exposed to the air more than the other samples, the gel structure changed more, owing to the solvent evaporation.

### 4.3. Thickness Measurements

The thickness values of the wet and dry PA6 membranes are gathered in Table 3. It can be seen that both values showed a downward trend with increasing gelation time. In fact, the increase in gelation time resulted in obtaining more compact structures with smaller pores. This result may be due to the evaporation of the solvent from membranes during the delay stage (Figure 1), in which the M_10_ membrane (i.e., the membrane with 10 min of gelation) had much more time for solvent evaporation. Furthermore, the spreading of the cast polymeric solution on the glass plate could be another reason for the thickness reduction, because of the low viscosity of the cast solution. Moreover, it can be seen that the thickness values of the wet membrane are higher than those for the dry membrane (Table 3). The water presence in the membrane pores in the wet form causes membrane swelling and, subsequently, an increase in membrane thickness. In addition, the membrane thickness measured by SEM is displayed in Figure 6, and the results follow the same trend as the results obtained by micrometer (Table 3). Furthermore, the thickness change was calculated taking into account the thickness of the PA6 membranes in wet (T_w_) and dry (T_d_) states (Equation (14)) [45,46].

(14) $$\mathrm{Thickness}\;\mathrm{change}\;(\%)=\left(\frac{T_{w}{-\;T}_{d}}{T_{d}}\right)$$

An opposite correlation between the gelation time and thickness change can be seen, i.e., with increasing gelation time, the membrane thickness change reveals a downward tendency (Table 3). The highest and lowest values were observed for the M_0_ and M_10_ membranes (around 8 and 3%, respectively). In fact, owing to the presence of bigger pores in the structure of the M_0_ membrane (Figure 5A), this membrane could absorb more water and, thus, swelled more than other samples. On the contrary, the formation of smaller pores in the case of the M_10_ membrane led to a decrease in the swelling ability of this membrane.

### 4.4. Contact Angle Measurement

**Figure 7:**
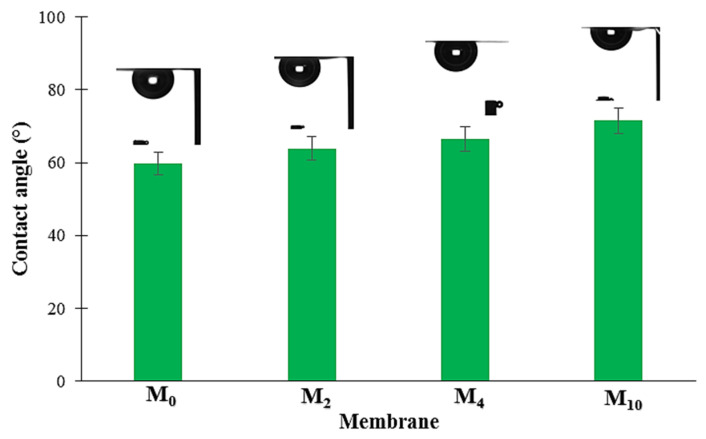
The contact angle of the PA6 membranes measured by captive bubble method.

The gelation time significantly influenced the physical features of the membranes (Figure 7 and Figure S1). The results of the contact angle analysis revealed that the increase in gelation time from 0 to 10 min caused an increase in the contact angle of the membranes (Figure 7). The M_0_ membrane had the lowest contact angle of 60 ± 4° (i.e., the highest hydrophilicity) whereas the M_10_ membrane possessed the highest contact angle of 72 ± 8° (i.e., the lowest hydrophilicity). Since no hydrophilic and hydrophobic additives were introduced to the casting solution, the surface roughness is the only factor for this observation (Table 2 and Figure S1). This trend confirms that there is a direct correlation between the gelation time and the membrane surface morphology (Figure S1), i.e., the higher the gelation time, the rougher the surface. The changes in the gel structure owing to the solvent evaporation during the delay stage led to the increase in roughness of the membranes.

### 4.5. Water Uptake (WU)

**Figure 8:**
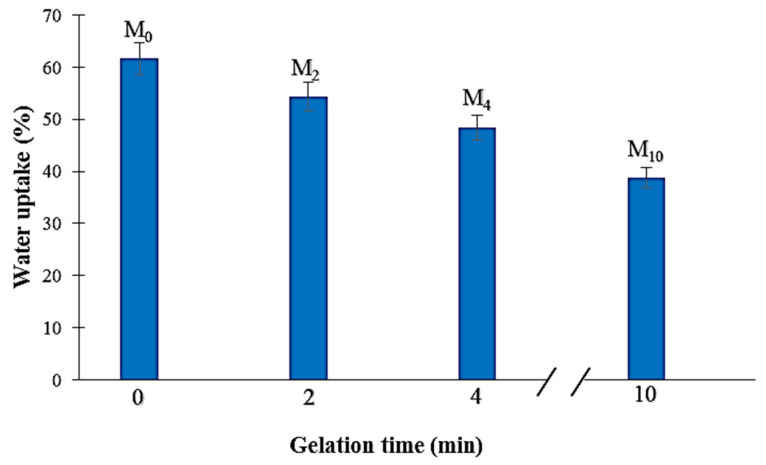
Water uptake of the obtained PA6 membranes as a function of the gelation time.

The results of the water uptake for PA6 membranes are shown in Figure 8. It can be seen that the increasing gelation time provokes a decrease in the membrane water uptake. The highest and lowest WU values were obtained for the M_0_ and M_10_ membranes (62 and 39%, respectively). In fact, the increase in gelation time led to a membrane pore size reduction (Figure 6) and, therefore, less water could be absorbed by the membrane (Figure 8).

### 4.6. Mean Pore Size and Porosity Measurements

The measured mean pore size, porosity, bubble point pressure, minimum and maximum pore size are presented in Table 4. The increase in the gelation time results in a decrease in the mean pore size−the smallest value (0.234 µm) is measured for the membrane with the highest gelation time (M_10_). The SEM images (Figure 5 and Figure 6) and membrane water uptake values (Figure 8) confirm these results as the membranes with denser structures are obtained with increasing gelation times. An average pore size of 0.710 and 0.234 µm was obtained for M_0_ and M_10_ membranes, respectively. Furthermore, the bubble point pressure was measured (Table 4). Generally, the bubble point pressure is the pressure at which the first air bubble passes through a wetted membrane. This pressure is correlated with the pore size of the membrane. It was observed that there was an opposite correlation between the bubble point pressure and the maximum pore size (i.e., more dense membranes (M_10_) had a higher bubble point pressure). This means that the denser membrane, the more pressure was needed to observe the first air bubble pass through the membrane. Moreover, an opposite correlation between the gelation time and membrane porosity was revealed (Table 4). It can be seen that an increase in the gelation time results in a reduction in porosity, as the highest porosity was obtained for the M_0_ membrane (around 72%) while the M_10_ membrane had the lowest porosity value (around 39%). As a result, less porous membranes can be obtained by increasing the time of gelation.

### 4.7. Mechanical Strength

**Figure 9:**
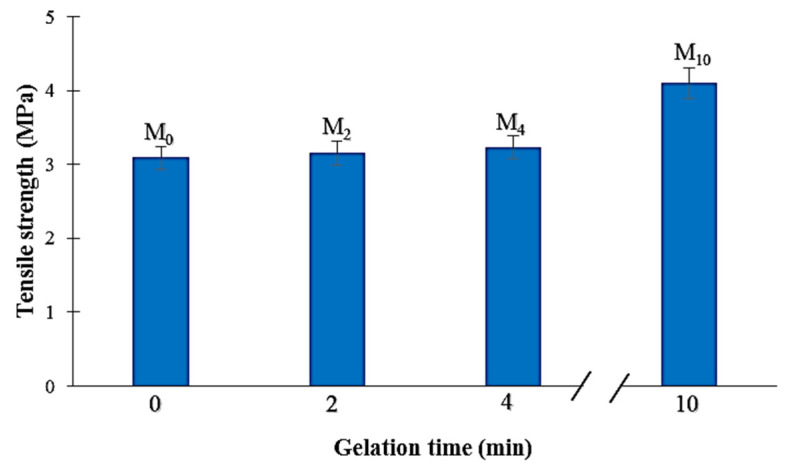
Tensile strength of the PA6 membranes with different gelation time.

As can be seen from Figure 9, the increase in gelation time from 0 to 4 min caused only negligible changes in the membrane tensile strength. This result is mostly due to the similar membrane structures obtained for these gelation times (Figure 5 and Figure 6). However, with further raising of the gelation time from 4 to 10 min, the membrane tensile strength increased from 3.2 to 4.1 MPa (Figure 9). Indeed, the smallest pores were obtained for the M_10_ membrane prepared with 10 min of gelation time (Figure 5D and Figure 6D). Therefore, it can be stated that a denser membrane structure with a smaller mean pore size (Table 4) can result in a higher membrane mechanical tensile strength.

### 4.8. Membrane Filtration Performance

**Figure 10:**
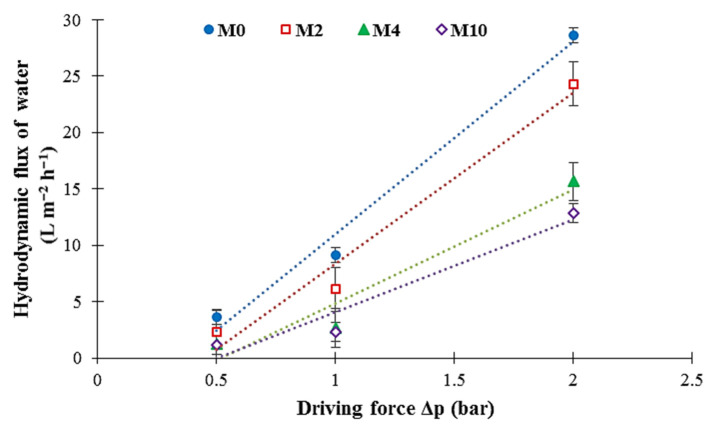
Influence of the operating pressure on the hydrodynamic water flux for different obtained membranes.

The hydrodynamic water flux of the PA6 membranes at three operating pressures (0.5, 1.0, and 2.0 bar) was measured by using the dead-end experimental setup (Figure 4). To measure the hydrodynamic water flux, the PA6 membrane was initially compressed at high pressure (3 bar) until the water flow was stabilized. The resultant PA6 membranes showed a considerable initial hydrodynamic water flux at 3 bar, i.e., the initial hydrodynamic water flux for the M_0_ sample was as high as 3500 L m^−2^ h^−1^. Figures S2–S5 show the changes in the hydrodynamic water flux versus the operating time. It can be seen that in all membrane samples, the hydrodynamic water flux reveals a downward tendency with increasing operating time, and then stabilizes at a constant value (Figures S2–S5). The main reason is that by applying pressure, the membrane starts to become compacted; thus, the flux reduces and remains stable after the completion of pore compression. The evolution of the hydrodynamic water flux of the PA6 membranes under different operation pressures is shown in Figure 10. The increase in the gelation time decreases the hydrodynamic water flux of the PA6 membranes. Generally, the hydrodynamic water flux is considerably affected by several factors, such as the mean pore size, porosity, morphology, and hydrophilicity of the membrane [36,47]. It is shown that the values of both mean pore size and porosity are reduced with an increase in gelation time (Table 4). Additionally, the SEM images show that by increasing the gelation time, a dense membrane with smaller pores is obtained (Figure 5 and Figure 6). The water contact angle results also reveal that the membrane hydrophilicity decreases with the increase in gelation time (Figure 7). As a result, the hydrodynamic water flux across the M_0_ membrane is higher than through the other membranes. Additionally, it can be seen that for each membrane sample, the hydrodynamic water flux increases linearly with the raising of the operation pressure from 0.5 to 2.0 bar (Figure 10). Under 2 bar of operating pressure, the M_0_ and M_10_ membranes have the highest and lowest hydrodynamic water fluxes of 28.6 and 12.9 L m^−2^ h^−1^, respectively.

**Figure 11:**
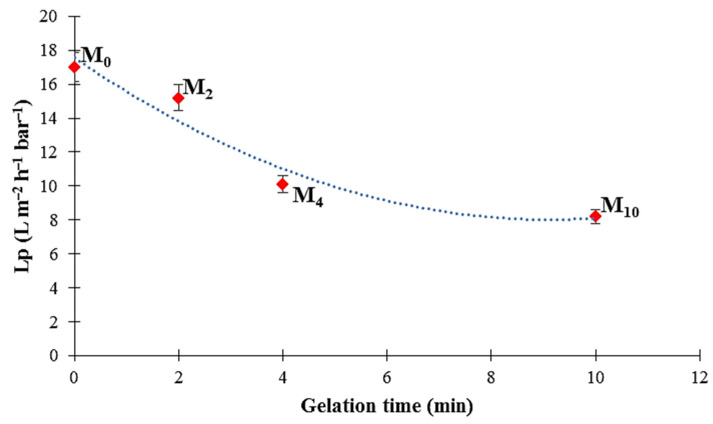
Membrane hydrodynamic permeability of the PA6 membranes as a function of the gelation time.

The membrane hydrodynamic permeability coefficient (L_p_) can be obtained from the slop of the hydrodynamic water flux curve as a function of the pressure. The obtained results of L_p_ at different gelation time are shown in Figure 11. In general, the high value of L_p_ is obtained for the membrane with more open pores, while the low L_p_ value characterizes a denser membrane [5]. The hydrodynamic permeability of the PA6 membranes showed a downward trend with increasing gelation time, and the highest and lowest L_p_ values are obtained for the M_0_ (more open) and M_10_ (denser) membrane samples, i.e., 17.0 and 8.2 L m^−2^ h^−1^ bar^−1^, respectively. According to the Hagen–Poiseuille equation (Equation (10)), the membrane hydrodynamic permeability correlates directly with the pore radius and membrane porosity. As presented in Table 4, with the increase in gelation time, the porosity and mean pore size of the membrane decreased. Consequently, the membrane hydrodynamic permeability was also reduced.

Here, the results of some researches regarding the hydrodynamic water flux and L_p_ are mentioned. Zheng et al. [48] prepared porous PA6 membranes. They improved the chemical and physical properties of the PA6 membranes by grafting ionic liquids onto PA6. The water flux of the pristine PA6 membrane was 1300 L m^−2^ h^−1^, while it was improved to 3400 L m^−2^ h^−1^ by grafting ionic liquid onto the PA6 membrane. In another study, Shin et al. [49] prepared polyethersulfone (PES)/2-methoxyethanol (2-ME)/n-methyl-2-pyrrolidone (NMP) microfiltration membranes. In that study, 2-methoxyethanol (2-ME) was used as an additive to improve the morphological properties of PES membranes. The resultant PES membranes showed good porosity in the range of 84–92%. Moreover, the mean pore sizes of the PES membranes were between 0.15 and 0.35 µm. The pure water flux of PES membranes was improved to 700 L m^−2^ h^−1^ using 2ME/NMP. Ferreira et al. [50] fabricated polyethersulfone (PES)/silver nanoparticles via a phase inversion method. They evaluated the effects of the polymer solution composition, the composition of the precipitation bath, and the gelation time before immersion on the morphological and transport features of the composite membranes. The highest and lowest values regarding the pure water permeability were 11,017 and 14.3 L m^−2^ h^−1^ bar^−1^, respectively. Fontão et al. [51] prepared polysiloxane-based SiOC membranes produced via a phase inversion tape casting process. The values regarding water permeation flux were between 6 and 55 m^3^ m^−2^ h^−1^ at 3 bar operating pressure. In another study, Woo et al. [52] fabricated poly(vinylidene fluoride) (PVDF) membranes by a phase inversion technique and invested the influence of the surface roughness on permeate flux. It was found that the permeate flux of membranes with smooth surfaces (145.89 L m^−2^ h^−1^) was 12% higher than membranes with rough surfaces (130.24 L m^−2^ h^−1^).

**Figure 12:**
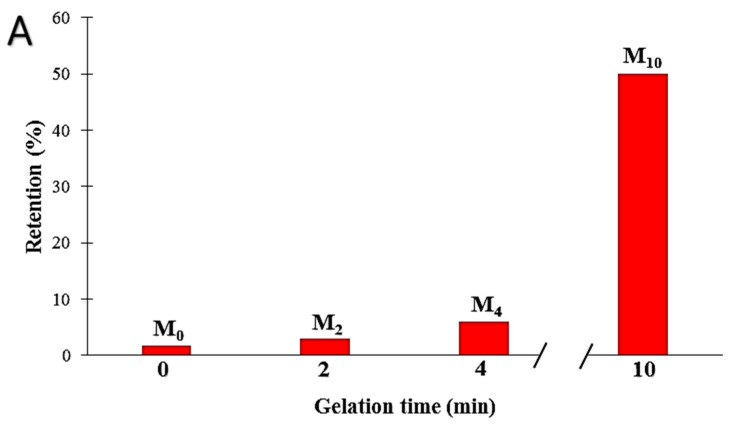

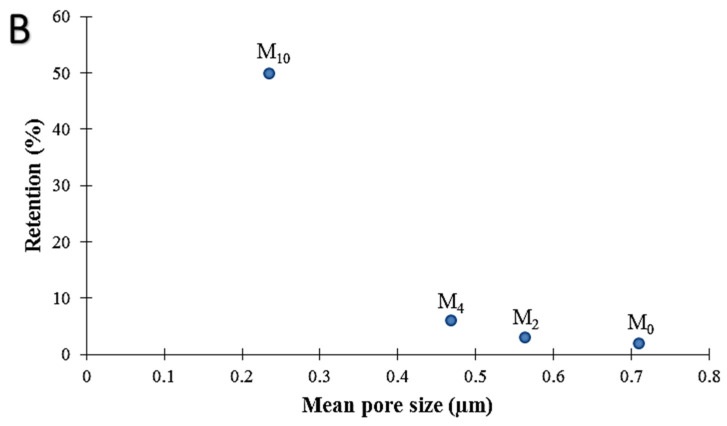
The influence of gelation time on retention ((**A**): retention vs. gelation time, (**B**): retention vs. mean pore size).

Moreover, as is observable in Figure 12A,B, by increasing the gelation time, the retention increased, and the highest value was observed for the M_10_ sample (around 50%) with the highest gelation time. It can be seen that the retention of the PA6 membranes has an opposite correlation with the mean pore size of the membrane (Figure 12B and Table 4). Therefore, it can be concluded that a higher gelation time leads to obtaining more dense membranes with a smaller mean pore size (MPS), leading to a higher membrane retention.

## 5. Conclusions

In this research, the influence of the gelation time on the physical and morphological properties of porous PA6 membranes was investigated. The SEM images (both surface and cross-section) revealed that the increase in gelation time significantly changed the membrane morphology. In addition, the increase in the gelation time decreased the thickness of dry and wet membranes. The AFM showed that the M_10_ membrane was the roughest membrane. Moreover, the membrane hydrophilicity reduced with the increase in gelation time. Besides, the gelation time increase provokes the formation of less porous membranes with smaller mean pore sizes and, thus, with increased mechanical stability. The highest measured hydrodynamic water flux and L_p_ values were 28.6 L m^−2^ h^−1^ (at Δp = 2 bar) and 17 L m^−2^ h^−1^ bar^−1^, respectively for the M_0_ membrane. On the other hand, the M_10_ membrane showed the lowest values under the same conditions, i.e., 12.9 L m^−2^ h^−1^ and 8.2 L m^−2^ h^−1^ bar^−1^, respectively. The obtained results demonstrated that not only the PA6 membrane morphology, but also the membrane transport properties are changed with the gelation time. The obtained results are promising for pressure-driven membrane processes, i.e., microfiltration, ultrafiltration, and nanofiltration.

## Figures and Tables

**Table 1:** Elaboration parameters of PA6 membranes.

Membrane	Gelation Time (min)
M_0_	0
M_2_	2
M_4_	4
M_10_	10

**Table 2:** Surface roughness results concerning PA6 membranes for the 5 µm × 5 µm scanned area.

Membrane	Gelation Time (min)	R_a_ (nm)	R_q_ (nm)
M_0_	0	154 ± 22	200 ± 34
M_2_	2	155 ± 32	195 ± 42
M_4_	4	166 ± 28	203 ± 31
M_10_	10	189 ± 53	240 ± 59

**Table 3:** Membrane thickness as a function of the gelation time.

Membrane	Gelation Time (min)	Thickness (µm)			Thickness Change (%)
		Wet	Dry	Measured by SEM	
M_0_	0	162 ± 8	150 ± 7	153	8
M_2_	2	150 ± 7	141 ± 15	145	6
M_4_	4	141 ± 7	134 ± 7	137	5
M_10_	10	124 ± 7	120 ± 16	119	3

**Table 4:** The influence of the gelation time on the membrane porosity and mean pore size.

Membrane	Porosity (%)	Mean Pore Size (µm) *	Minimum Pore Size (µm) *	Maximum Pore Size (µm) *	Bubble Point Pressure (bar) *
M_0_	72 ± 2	0.710 ± 0.060	0.166	2.832	0.226
M_2_	59 ± 1	0.563 ± 0.036	0.364	1.449	0.442
M_4_	54 ± 2	0.468 ± 0.023	0.223	1.036	0.618
M_10_	39 ± 4	0.234 ± 0.019	0.153	0.960	0.667

*—The applied liquid was Porefil with a surface tension of 16 mN m^−1^.

## Data Availability

Data associated with this research are mentioned in the article.

## Supplementary Materials

The following supporting information can be downloaded at: https://www.mdpi.com/article/10.3390/membranes12030315/s1, Figure S1: Surface morphology of PA6 membranes with different gelation time for 5 µm × 5 µm scanned area. Figure S2: Hydrodynamic water flux of M0 membrane (gelation time 0 min) at three operating pressures. Figure S3: Hydrodynamic water flux of M2 membrane (gelation time 2 min) at three operating pressures. Figure S4: Hydrodynamic water flux of M4 membrane (gelation time 4 min) at three operation pressures. Figure S5: Hydrodynamic water flux of M10 membrane (gelation time 10 min) at three operating pressures.

## Abbreviations

R_a_ (nm)	Average roughness
AFM	Atomic force microscopy
CA (deg)	Contact angle
T_d_ (µm)	Dry thickness
MPS (µm)	Mean pore size
L_p_ (L m^−2^ h^−1^ bar^−1^)	Membrane hydrodynamic permeability
NP	Nanoparticle
PA6	Polyamide-6
PEG	Polyethylene glycol
ε (%)	Porosity
RH (%)	Relative humidity
R_q_ (nm)	Root mean square
SEM	Scanning electron microscopy
WU (%)	Water uptake
T_w_ (µm)	Wet thickness
