# Supplementary material for: Fabrication of Polyamide-6 Membranes—The Effect of Gelation Time towards Their Morphological, Physical and Transport Properties
Source: Membranes (Basel). 2022 Mar 10;12(3):315. doi: 10.3390/membranes12030315 (PMC8949474; doi:10.3390/membranes12030315)
Supplement: Supplementary file 1 [file membranes-12-00315-s001.zip › membranes-1530425-supplementary.pdf]

membranes

Supplementary Material

Fabrication of Polyamide-6 Membranes—The Effect of Gelation Time towards Their Morphological, Physical, and Transport Properties

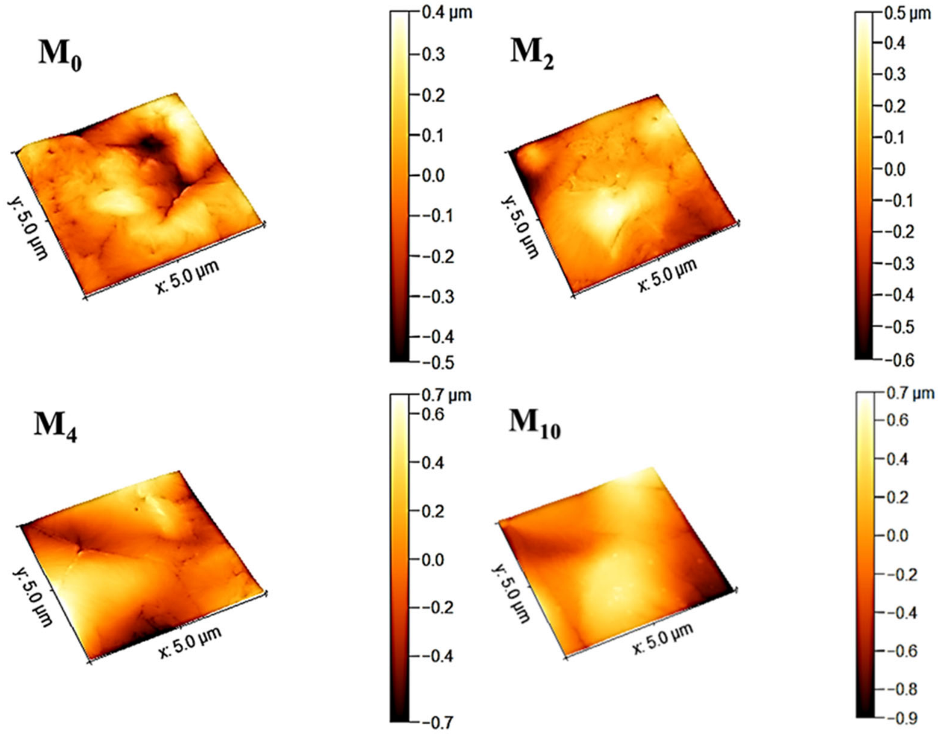

Figure S1. Surface morphology of PA6 membranes with different gelation time for 5 µm × 5 µm scanned area.

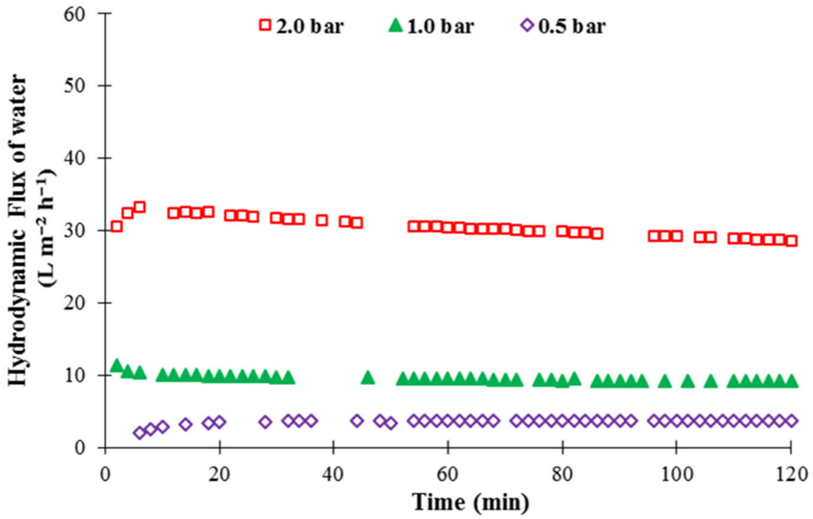

Figure S2. Hydrodynamic water flux of M_0 membrane (gelation time 0 min) at three operating pressures.

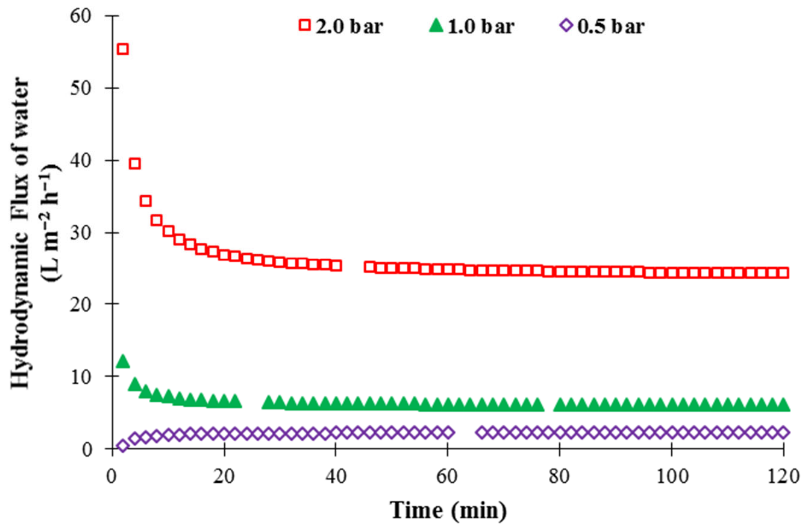

Figure S3. Hydrodynamic water flux of M_2 membrane (gelation time 2 min) at three operating pressures.

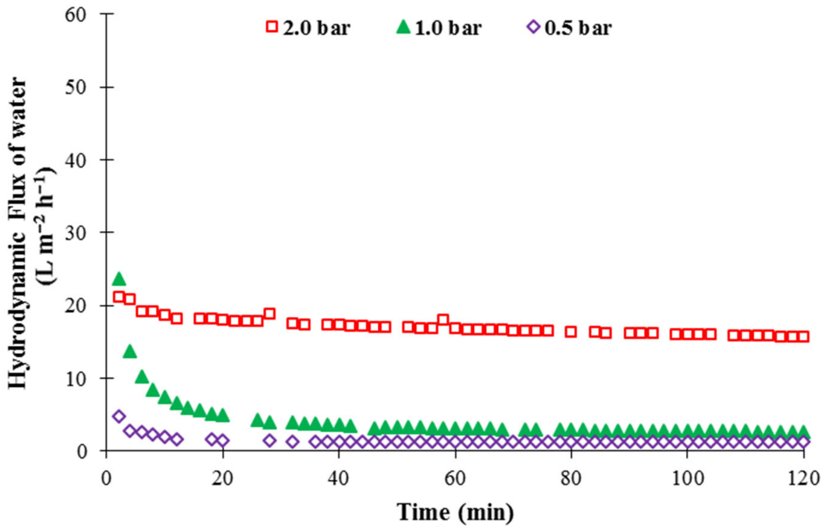

Figure S4. Hydrodynamic water flux of M_4 membrane (gelation time 4 min) at three operation pressures.

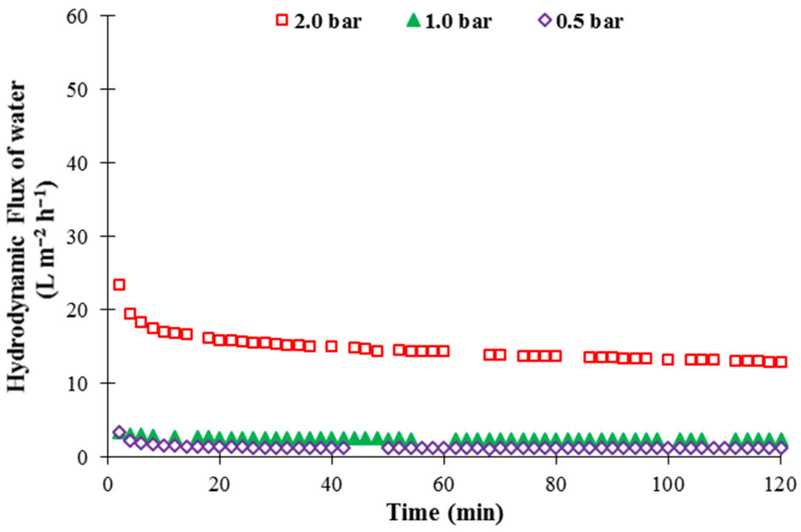

Figure S5. Hydrodynamic water flux of M_{10} membrane (gelation time 10 min) at three operating pressures.
